# Pleural fluid prealbumin and C-reactive protein in the differential diagnosis of infectious and malignant pleural effusions

**DOI:** 10.3892/etm.2014.1503

**Published:** 2014-01-27

**Authors:** QIAOYING JI, BIFEI HUANG, MAOFENG WANG, ZHAOXIANG REN, SHA ZHANG, YONGJUN ZHANG, LIJIAN SHENG, YAYAO YU, JINWEN JIANG, DEBAO CHEN, JUN YING, JIONG YU, LIUYI QIU, RUGEN WAN, WEIMIN LI

**Affiliations:** 1 Department of Respiratory, Affiliated Dongyang Hospital of Wenzhou Medical University, Dongyang, Zhejiang 322100, P.R. China; 2 Pathology Center, Affiliated Dongyang Hospital of Wenzhou Medical University, Dongyang, Zhejiang 322100, P.R. China; 3 Department of Clinical Laboratory Medicine, Affiliated Dongyang Hospital of Wenzhou Medical University, Dongyang, Zhejiang 322100, P.R. China; 4 School of Laboratory Medicine, Wenzhou Medical University, Wenzhou, Zhejiang 325035, P.R. China; 5 State Key Laboratory for Diagnosis and Treatment of Infectious Diseases, First Affiliated Hospital, School of Medicine, Zhejiang University, Hangzhou, Zhejiang 310003, P.R. China; 6 Department of Internal Medicine, Affiliated Dongyang Hospital of Wenzhou Medical University, Dongyang, Zhejiang 322100, P.R. China

**Keywords:** prealbumin, C-reactive protein, pleural effusion

## Abstract

Clinical history and physical examination are helpful in indicating the potential causes of pleural effusions (PEs). However, the accurate diagnosis and establishment of the causes of PE is an ongoing challenge in daily clinical practice. The primary aim of this study was to distinguish between infectious PE and malignant PE (MPE) by measuring two major acute phase response biomarkers: prealbumin (PA) and C-reactive protein (CRP). The study was a prospective trial involving 151 patients who were diagnosed with infectious PE or MPE. Patients with infectious PE were divided into two subgroups: tuberculous PE (TBPE) and parapneumonic PE (PNPE). A further 58 patients with PEs that showed no evidence of MPE, TBPE or PNPE were classified as the chronic non-specific PE (NSPE) group. Demographic characteristics and pleural fluids of the subjects were collected consecutively. The discriminative properties of pleural fluid routine biochemistries, and PA and CRP were evaluated. PA, CRP and classical fluid parameters were also applied to classify patients with infectious PE and MPE. Receiver operating characteristics (ROC) analysis established the cutoffs of PA and CRP for discriminating between groups. Pleural fluid PA levels were significantly higher in the MPE group (n=47) than in the infectious PE group (n=104). Pleural fluid CRP levels were significantly higher in the infectious PE group than in the MPE group. Pleural fluid PA levels were identified to be moderately negatively correlated with CRP levels in the MPE group, with a statistically significant correlation coefficient of −0.352. The ROC curve showed that the sensitivity and specificity of PA for the diagnosis of MPE were 0.851 and 0.548, respectively, at the cutoff of 28.3 mg/l. The area under the curve (AUC) was 0.784 (95% CI, 0.707–0.861). Using CRP as a diagnostic parameter resulted in an comparable AUC of 0.810 (95% CI, 0.736–0.885), at the cutoff of 35.2 mg/l. Combinations of PA and CRP resulted in incrementally discriminating values for MPE, with a sensitivity of 0.617 and a specificity of 0.903. The measurement of PA and CRP levels in pleural fluid may be a useful adjunctive test in PE, as a potential differentiator between infectious PE and MPE.

## Introduction

Approximately 1.5 million individuals develop pleural effusions (PEs) in the USA each year (1). PE is the abnormal accumulation of fluid between the two layers of pleura resulting from disruption of the homeostatic forces that control the flow into and out of the area. The accumulation of PEs is associated with numerous medical conditions; the most common conditions that cause PE are cardiac failure, pneumonia and malignant neoplasm disease (2–4). Diagnosis of a PE begins with obtaining the patient’s clinical history and performing a physical examination, and is followed by chest radiography and the analysis of pleural fluid in appropriate instances. Thoracentesis is routinely performed on patients with PE; the aspirated pleural fluid is usually sent for biochemical, microbiological and cytological analyses, with the first being readily available for decision-making (5). In clinical practice, PEs are classified as transudates or exudates, according to the biochemical characteristics of the fluid. Grouping PEs into transudates and exudates does not attach a specific label to the disease. Certain experts, including Light (1), have recommended focusing research on the identification of specific pleural disease markers rather than spending excessive time and resources in transudate -exudate differentiation. For further testing, malignant and benign effusions are often a diagnostic challenge. The diagnosis of a PE is not always straightforward, particularly when patients have coexisting heart failure, infection (6,7) or malignancy (5,8–10). Conventional leukocyte counts, effusion cell and differential counts, and Light’s criteria do not provide adequate information (11–14). Microbiological studies may provide more definitive results; however, the diagnosis yield rate is ~60% and the long turnaround time may result in a delayed diagnosis (3,5,15). Cytological examination of pleural fluid is a convenient and relatively efficient method for establishing the diagnosis of pleural malignancy. However, pleural fluid cytology is positive in only 50% of cases, leading to the requirement for further diagnostic tests (16). In clinical practice a variety of laboratory tests are in use for the differential diagnosis of PE; nevertheless, the efficiency of these measurements is not always sufficient to establish a diagnosis (1,17). Thus, the requirement for biomarkers that may aid in this differentiation is imperative.

In recent years, the development of disease-specific diagnostic biomarkers for common causes of PE has become an area of active research. PEs that are secondary to malignancy or infection usually have similar biochemical profiles, but certain biomarkers may have potential diagnostic value. These include PA (18) and CRP (14,15,19), which have been recently used to improve the differentiation between benign PE (BPE) and MPE. CRP is an acute-phase reactant produced primarily by hepatocytes, whose production is induced by systemic inflammation of either infectious or noninfectious origin (14,15). PA is a carrier of thyroxin and retinol (vitamin A), is a well-known negative acute-phase protein and is downregulated during inflammation (18).

At present, none of the proposed disease-specific biomarkers have gained widespread acceptance (5). Few studies have investigated the diagnostic role of pleural fluid PA (18) and CRP (14) in the etiology of PE, so there is little evidence available and the external validity has not been adequately analyzed. Additional studies are necessary to evaluate the roles of these biomarkers, individually and in combination, in the diagnosis of PE. Measurement of these two biomarkers is simple, rapid and cheap in the laboratory. Therefore, the primary aim of this study was to distinguish between infectious PE and MPE by measuring the levels of the two major acute phase response biomarkers, PA and CRP.

## Materials and methods

### Ethics statement

Ethical approval for this study was granted from the Affiliated Dongyang Hospital of Wenzhou Medical University Human Investigation Ethics Committee (Dongyang, China) and all patients provided written informed consent for enrolment into the study.

### Subjects and sample collection

PE specimens were prospectively collected from 209 patients who presented at the Affiliated Dongyang Hospital of Wenzhou Medical University between September 2012 and May 2013. The etiology of PE was based on accepted criteria (20); MPE was diagnosed by the identification of malignant cells in pleural fluid or pleural biopsy during cytological or histological examinations. Patients with infectious PE were divided into two subgroups: tuberculous PE (TBPE) and parapneumonic PE (PNPE). TBPE was diagnosed with the presence of positive stain or culture for *Mycobacterium tuberculosis* in the pleural fluid, sputum or pleural biopsy, or the presence of typical caseating granulomas in the pleural biopsy. PNPE was characterized by any PE associated with pneumonia and a response to antibiotics; patients with pleural empyema were also included in this group. Patients with PE that showed no evidence of MPE, TBPE or PNPE were classified as chronic non-specific PE (NSPE). Exclusion criteria included hyperlipidemia, coronary heart disease, central nervous system diseases, such as intracranial tumors or Alzheimer’s disease, cholelithiasis and liver disease. The baseline demographic characteristics of the patients are summarized in Table I. Samples of the pleural fluid from the participants were frozen at −80°C until analysis.

### Biochemical analyses and differential cell counts

The demographic variables, the values of biochemical parameters in the pleural fluid and the levels of PA and CRP were analyzed prior to the start of treatment. The biochemical parameters were as follows: differential cell counts, pH, total proteins, lactate dehydrogenase (LDH), glucose, total cholesterol (TC), triglycerides (TG) and adenosine deaminase (ADA). In addition, if a patient had been submitted to repeated thoracentesis, only the results of the first were considered. The biomarker levels in the pleural fluid were determined using a Hitachi 7600 clinical analyzer (Hitachi, Tokyo, Japan). The pH readings were obtained using a selective electrode in various standard blood-gas analyzers (Radiometer, Bronshoj, Denmark). Differential cell counts were detected using a Sysmex XE-2100 Automated Hematology system (Sysmex, Kobe, Japan). In addition, cytological examination of PEs on pleural fluid smears was performed following Wright’s staining.

### Statistical analysis

Results are presented as mean ± standard deviation or median and interquartile range (IQR), depending on the distribution. Univariate comparisons of continuous variables were performed using an unpaired Student’s t-test for normally distributed data, or nonparametric Mann-Whitney U test for non-normally distributed variables. For multiple comparisons of several groups, ANOVA or a Kruskal-Wallis test were performed. For comparing categorical data, a chi-square test was performed. Spearman’s rank correlation coefficient was used to investigate the association between PA and CRP in each group. The receiver operating characteristics (ROC) analysis was used to determine the optimum cutoff value for the studied diagnostic markers. Furthermore, the accuracy of pleural fluid biomarkers in distinguishing between infectious and malignant PE was established by calculating sensitivity, specificity, positive predictive values (PPVs) and negative predictive values (NPVs). P<0.05 was considered to indicate a statistically significant result. All statistical calculations were performed using the SPSS 13.0 for Windows (SPSS, Inc., Chicago, IL, USA).

## Results

### Demographic characteristics of the study subjects

The study subjects included 122 men and 87 women with a mean age of 56 years. One hundred and three patients had a history of smoking and 37 had a previous history of cancer (17 MPE patients, 6 TBPE patients, 8 PNPE patients and 6 NSPE patients). Among the 47 patients with MPE, 22 (47%) had a positive cytology in the initial thoracentesis examination; in the remaining 25 patients, MPE was finally confirmed by repeated cytological analysis or histological examination of pleural biopsy. Of the 25 patients with an initial negative cytology, eight had positive cytology in the second thoracentesis examination, 10 had positive cytology in the third thoracentesis, four had positive cytology in the fourth thoracentesis and three achieved a positive histology result in the pleural biopsy. The demographic characteristics of the study subjects are presented in Table I. The mean age of the MPE group was higher than those of the TBPE, PNPE and NSPE groups. There were no significant differences among the four groups with regard to subject gender ratios.

### Basic characteristics of pleural fluid samples

The basic characteristics of the pleural fluid samples are recorded in Table II. A Kruskal-Wallis test was employed to compare pleural markers among the four groups (P<0.05) and this was followed by the Mann-Whitney U test to evaluate the differences between each two groups. The median leukocyte concentration in the infectious PE group was higher than that in the MPE group. The levels of neutrophils (%) in the PNPE group were significantly higher than those in the other groups, and the levels of lymphocytes (%) and ADA in the TBPE group were significantly higher than those in the other groups. No significant differences in the levels of glucose and LDH were identified between the MPE and infectious PE groups. The levels of albumin (ALB) and TC in the MPE and TBPE groups were significantly higher than those in the other two groups. The comparison of conventional pleural markers between infectious PEs and MPEs presented similar results to those in previous reports (14,15). However, the levels of TG were significantly different among the four groups as revealed by the Mann-Whitney U test.

### Levels of PA and CRP in pleural fluid

**Figure 1:**
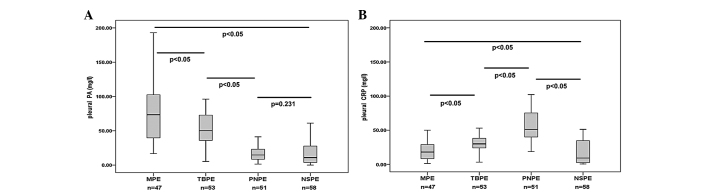
Levels of (A) PA and (B) CRP in pleural fluid. The box indicates the lower and upper quartiles and the central line marks the median. The points at the end of the whiskers represent the range of the values. PA, prealbumin; CRP, C-reactive protein; MPE, malignant pleural effusion; TBPE, tuberculous pleural effusion; PNPE, parapneumonic pleural effusion; NSPE, non-specific pleural effusion.

The median level of PA in the pleural fluid was highest in the MPE group (73 mg/l), followed by the TBPE (50 mg/l), PNPE (15 mg/l) and NSPE (11 mg/l) groups; the difference in pleural fluid PA levels between the PNPE and NSPE groups was not observed to be significant (Fig. 1A). The median level of CRP in the pleural fluid was highest in the PNPE group (52 mg/l), followed by the TBPE (30 mg/l), MPE (18 mg/l) and NSPE (9 mg/l) groups. No significant differences were observed between pleural fluid CRP levels in the MPE and NSPE groups (Fig. 1B).

### Correlation between PA and CRP in pleural fluid

**Figure 2:**
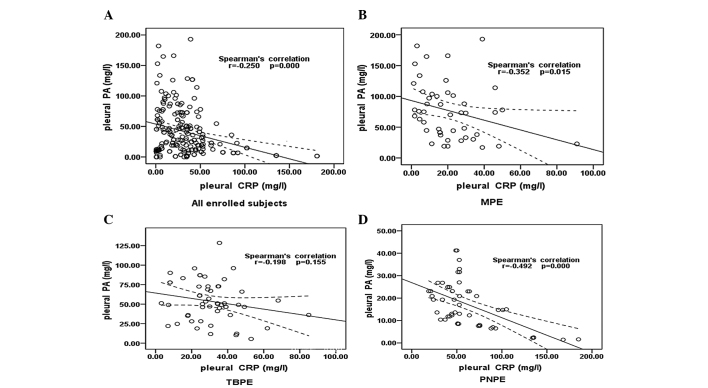
Correlation between PA and CRP levels in pleural fluid in (A) all enrolled subjects, (B) the MPE group; (C) the TBPE group and (D) the PNPE group. PA, prealbumin; CRP, C-reactive protein; MPE, malignant pleural effusion; TBPE, tuberculous pleural effusion; PNPE, parapneumonic pleural effusion.

Spearman’s correlation test was performed to analyze the correlation between PA and CRP. In the correlation analysis of patients with PEs (Fig. 2), the levels of pleural fluid PA correlated with the pleural fluid CRP levels in the enrolled subjects with a statistically significant correlation coefficient of −0.250 (Fig. 2A). Spearman’s correlation analysis was also conducted to investigate the correlation between pleural fluid PA and CRP in infectious PE and MPE. A statistically significant negative correlation was identified (r=−0.352; P=0.015) in the MPE group (Fig. 2B). However, there was a non-statistically significant negative correlation (r=−0.198; P=0.155) in the TBPE group (Fig. 2C). Pleural fluid PA levels were significantly negatively correlated with pleural fluid CRP levels with a correlation coefficient of −0.492 in the PNPE group (Fig. 2D).

### Diagnostic values of PA and CRP in pleural fluid

**Figure 3:**
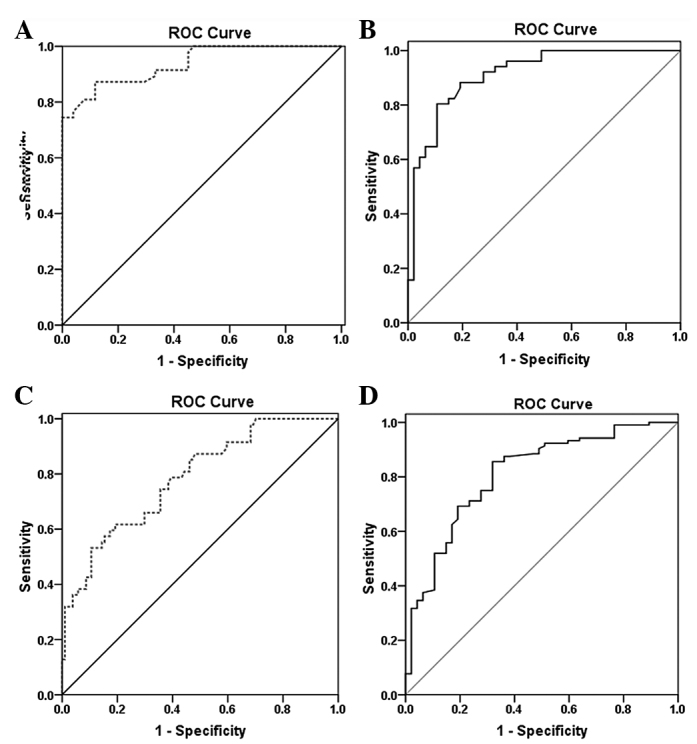
ROC curve analyses for the diagnostic values of PA and CRP in pleural fluid. Discrimination between MPE and PNPE using (A) PA and (B) CRP, and discriminate between infectious PE and MPE using (C) PA and (D) CRP.. ROC, receiver operating characteristics; PA, prealbumin; CRP, C-reactive protein; PE, pleural effusion; MPE, malignant pleural effusion; PNPE, parapneumonic pleural effusion.

In ROC curve analysis to discriminate between MPE and PNPE (Fig. 3A and B), pleural fluid PA and CRP levels were demonstrated to have high diagnostic accuracy for discriminating MPE and PNPE. The AUC of pleural fluid CRP [0.913 (95% CI, 0.857–0.969)] tended to be smaller than that of pleural fluid PA [0.937 (95% CI, 0.891–0.982)]. However, the overlapping 95% CI values for the two markers indicated that neither was superior. If the analysis was expanded to patients with infectious PE and MPE (Fig. 3C and D), the diagnostic accuracy of pleural fluid PA (AUC, 0.784; 95% CI, 0.707–0.861) and CRP (AUC, 0.810; 95% CI, 0.736–0.885) for discriminating between infectious PE and MPE tended to be lower than the diagnostic accuracy for discriminating between MPE and PNPE. For the analysis, the cutoffs for the pleural fluid PA and CRP levels were 28.3 and 35.2 mg/l, respectively.

### Predictors of MPE

The performances of pleural fluid PA and CRP at the optimum cutoff values for differentiating between infectious and malignant PE are listed in Table III. CRP exhibited superior discriminative properties, yielding an AUC ≥0.80. PA and CRP were potential predictors of MPE due to low PPVs. The sensitivity, specificity, PPV and NPV for MPE when the values for PA and CRP exceeded or did not meet the suggested diagnostic cutoff values were calculated. If both tests were positive, the specificity increased to 0.903. In particular, the criteria of pleural fluid PA >28.3 mg/l and CRP <35.2 mg/l had the highest PPV. However, if either test was positive, the sensitivity for detecting MPE increased to 0.936. The corresponding PPVs, NPVs and AUCs are shown in Table III.

## Discussion

Clinical history and physical examination are helpful for indicating the potential causes of PEs. However, the accurate diagnosis and establishment of the causes of PE is an ongoing challenge in daily clinical practice, despite the wide variety of laboratory tests and complementary studies that been carried out for the differential diagnosis of PE (2,9,21,22). The analysis of soluble biomarkers from effusions may be a useful adjunctive diagnostic tool. Considerable effort has been made to develop a simple, inexpensive and noninvasive method for distinguishing different types of PE in laboratory and clinical settings (23–27). No standard biochemical approach has yet been established. Ideal biomarkers should be easily measured at a reasonable cost (analytical validity), sensitive and specific to the disease state being examined, and aid in decision-making (clinical usefulness) (28). However, few fulfill these three criteria sufficiently to be used clinically. Biomarkers such as PA and CRP have potential diagnostic value due to their simple, rapid and cheap detection in the clinical laboratory. The present study indicates that pleural fluid PA and CRP are potentially powerful differential diagnostic tools for infectious PE and MPE. In particular, these data are widely available (as a part of the clinical detection conducted in the laboratory) to the clinician at a low cost. To the best of our knowledge, this is the first study to associate PA and CRP with infectious and malignant PE.

Unlike previous studies (29,30), which reported that the concentration of PA was lower in the sera of cancer patients than in that of normal individuals, the present study indicates that the median pleural fluid PA level was higher in the MPE group (73 mg/l) compared with the levels observed in patients with PE of other etiologies. However, no significant differences between pleural fluid PA levels were identified between the PNPE and NSPE groups; therefore, PA did not demonstrate any diagnostic value for patients with PNPE and NSPE. Wang *et al* (18) reported similar findings from a clinical trial of patients with PE from lung cancer and benign inflammatory disease; PA was overexpressed in PEs from patients with lung cancer, but not from patients with benign inflammatory disease. The median pleural fluid CRP level was higher in the PNPE group (52 mg/l) compared with the levels observed in patients with PE of other etiologies. However, there were no significant differences between pleural fluid CRP levels in the MPE and NSPE groups, so CRP did not demonstrate any diagnostic value for patients with MPE and NSPE. Similar independent associations have been revealed by previous studies evaluating the role of pleural fluid CRP (15,17,19); the highest pleural fluid CRP levels were observed in patients with PNPE. However, the findings of the present study conflict with the observations of Botana-Rial *et al* (14), who found no correlation between CRP values in TBPE and MPE patients. Porcel *et al* (15) differentiated TBPE from MPE when CRP levels in pleural fluid were >20 mg/l. The results of the present study are similar, as the CRP levels in the pleural fluid were higher in the TBPE group than in the MPE group (30 vs. 18 mg/l). Although the precise reasons for the difference in the reported associations remain speculative, it is plausible that differences in population characteristics, sample size and the different detection methods used, may have been responsible.

PA is a nutritional marker used to evaluate recent nutritional status with a short half-life and a rapid synthesis rate, and it is also a negative acute-phase protein and inversely associated with inflammation (31). CRP is one of the widely used biomarkers for monitoring the course of infection and inflammation. The results of the current study indicate that there was a weak negative correlation between pleural fluid PA and CRP levels in the enrolled subjects. Pinilla *et al* (32) demonstrated a strong correlation between the ratio of CRP to PA and the severity of organ dysfunction in critically ill patients. The present study also investigated the correlation between pleural fluid PA and CRP levels in MPEs and PNPEs. There was a statistically significant negative correlation (r=−0.352) in the MPE group and a statistically significant negative correlation with a correlation coefficient of −0.492 in the PNPE group. It is uncertain whether the high levels of PA in MPE are the consequence of the pathological processes that take place or whether the increase in PA participates in the induction of MPE. The pathophysiological significance of the observations in the present study may be investigated through serial measurements and further assessment of these markers in a larger prospective study. These data provide evidence supporting the measurement of CRP and PA levels as an inexpensive and useful tool in the evaluation of PE.

The diagnostic sensitivity, specificity and AUCs of PA levels in comparison with CRP levels for the diagnosis and differential diagnosis of different types of PE were fully investigated in the present study. It was observed that threshold levels of PA and CRP without high PPV were insufficient for the identification of MPE patients. For example, at the cutoff 28.3 mg/l, the sensitivity and specificity of PA were 0.851 and 0.548, respectively, for the diagnosis of MPE. The AUC was 0.784 and the PPV was 0.460. Used in a clinical situation, this means that 54% of subjects would have a false positive result, while 15% of the subjects with MPE would be missed. Choosing a low PA cutoff point for clinical practice would lead to unnecessary tumor treatment for a significant number of patients. Increasing the cutoff decreases the false positive rate and improves the specificity of the test measurement. It may be concluded that neither of the two variables is satisfactory for the differential diagnosis of different types of PE. Although the data have been shown to be of differential value, they are of limited use. The most important limitation of using individual fluid parameters in the discrimination process, namely the lack of test sensitivity, may be resolved by using either biomarker. For instance, the finding in the pleural fluid of a CRP <35.2 mg/l or a PA>28.3 mg/l characterized MPE with a sensitivity of 93.6%, thus increasing the sensitivity of each individual analysis by 9%. Higher PPVs were achieved by combining changes in PA and CRP, but still at the expense of sensitivity. For example, CRP <35.2 mg/l and PA >28.3 mg/l resulted in a PPV of 0.743, but the corresponding sensitivity was 0.617. Similar values were observed with other combinations of different threshold levels.

The limitations of this study require consideration. A relatively small sample size may cause a certain bias of the results. Patients with MPE from different primary tumors were included, which may influence the detection result of these molecular markers. In addition, the lack of differentiation between complicated and uncomplicated PNPE may affect the findings. Despite the potential biases, the authors suggest that the strong statistical significance of the results allows for optimism regarding PA and CRP as potential biomarkers for diagnosing PE.

In conclusion, the use of pleural fluid PA and CRP levels for the differential diagnosis of infectious and malignant PE was assessed. The measurement of pleural fluid PA and CRP levels may be a useful adjunctive test in PE, as a potential differentiator between infectious PE and MPE.

## Figures and Tables

**Table I:** Subject characteristics.

Group	Enrolled subjects (n)	Age (years) (mean ± SD)	Male gender n (%)
MPE	47	70±11	29 (61.7)^b^
Lung adenocarcinoma	22	71±15	13 (59.1)
Mesothelioma	5	62±14	3 (60.0)
Other lung carcinoma	7	62±19	4 (57.1)
Metastatic carcinoma	13	54±17	9 (69.2)
TBPE	53	54±22^a^	33 (62.3)^b^
PNPE	51	50±16^a^	28 (54.9)^b^
NSPE	58	53±17^a^	32 (55.2)^b^
Total	209	56±16	122 (58.4)

SD, standard deviation; MPE, malignant pleural effusion; TBPE, tuberculous pleural effusion; PNPE, parapneumonic pleural effusion; NSPE, chronic non-specific pleural effusion.

a Mean ages were not significantly (P>0.05) different among these three groups.

b Gender ratios were not significantly (P>0.05) different among these four groups.

**Table II:** Basic characteristics of pleural fluid samples.

Pleural markers	MPE (n=47)	TBPE (n=53)	PNPE (n=51)	NSPE (n=58)	P-value^a^
Leukocytes (/μl)	1360 (500–2100)^d^	3100 (1930–3900)^c^	3000 (1220–4600)^c^	450 (225–1100)^e^	<0.01
Neutrophils (%)	10 (6–20)^d^	3 (2–10)^e^	82 (65–91)^c^	9 (5–25)^d^	<0.01
Lymphocytes (%)	60 (32–78)^d^	78 (62–89)^c^	11 (3–22)^f^	30 (15–41)^e^	<0.01^b^
Glucose (mmol/l)	6.0 (3.8–7.6)^d^	5.1 (4.4–5.9)^e^	6.4 (5.5–8.0)^d^	8.1 (7.4–9.1)^c^	<0.01
Proteins (g/l)	43 (31–49)^d^	49 (43–53)^c^	26 (17–41)^e^	22 (17–31)^e^	<0.01
ALB (g/l)	30 (19–35)^c^	29 (25–32)^c^	15 (10–25)^d^	14 (12–22)^d^	<0.01
TC (mmol/l)	2.01 (1.29–2.65)^c^	2.23 (1.85–2.49)^c^	0.94 (0.57–1.40)^d^	0.75 (0.49–1.27)^d^	<0.01
TG (mmol/l)	0.31 (0.17–0.48)^d^	0.37 (0.25–0.53)^c^	0.21 (0.16–0.35)^e^	0.12 (0.07–0.20)^f^	<0.01^b^
LDH (IU/l)	361 (162–486)^c^	374 (285–518)^c^	206 (139–986)^c^	122 (78–189)^d^	<0.01
ADA (U/l)	12 (7–30)^d^	64 (43–87)^c^	8 (5–25)^d^	7 (5–23)^d^	<0.01
pH	7.41 (7.30–7.48)^c^	7.38 (7.29–7.49)^c^	7.28 (7.12–7.44)^d^	7.43 (7.36–7.51)^c^	<0.01

Measurement data are expressed as median (IQR). IQR, interquartile range; ALB, albumin; TC, total cholesterol; TG, triglycerides; LDH, lactate dehydrogenase; ADA, adenosine deaminase; MPE, malignant pleural effusion; TBPE, tuberculous pleural effusion; PNPE, parapneumonic pleural effusion; NSPE, chronic non-specific pleural effusion.

a Significance level of Kruskal-Wallis test.

b Significant differences among the four groups by Mann-Whitney U test.

c–f Pleural marker levels significantly (P<0.05) decreased in the order c>d>e>f, and results on the same row labeled with the same letters are indicated to have no significant differences.

**Table III:** Pleural fluid PA and CRP levels for the diagnosis of infectious and malignant PEs.

Biomarkers	Cutoff	Sensitivity	Specificity	PPV	NPV	AUC	P-value
PA	>28.3 mg/l	0.851	0.548	0.460	0.890	0.784	<0.05
CRP	<35.2 mg/l	0.856	0.680	0.680	0.856	0.810	<0.05
PA or CRP	PA >28.3 mg/l or CRP <35.2 mg/l	0.936	0.490	0.454	0.944	-	-
PA and CRP	PA >28.3 mg/l and CRP <35.2 mg/l	0.617	0.903	0.743	0.854	-	-

PA, prealbumin; CRP, C-reactive protein; PE, pleural effusion; PPV, positive predictive value; NPV, negative predictive value; AUC, area under the curve.
